# Redox Reprogramming of the Diseased Liver by Dietary Flavonoids: From Molecular Signalling to Gut–Liver Crosstalk

**DOI:** 10.3390/biology15080625

**Published:** 2026-04-16

**Authors:** Shivani Dogra, Ananta Prasad Arukha, Bhupendra Koul, Muhammad Fazle Rabbee

**Affiliations:** 1 Department of Microbiology, School of Bioengineering and Biosciences, Lovely Professional University, Phagwara 144411, India; 2 Department of Infectious Disease and Immunology, College of Veterinary Medicine, University of Florida, Gainesville, FL 32110, USA; 3 Department of Biotechnology, School of Bioengineering and Biosciences, Lovely Professional University, Phagwara 144411, India; 4 Department of Biotechnology, Yeungnam University, Gyeongsan 38541, Republic of Korea

**Keywords:** redox signalling, dietary flavonoids, gut-liver axis, Nrf2, oxidative stress, hepatoprotection, mitochondrial dysfunction

## Abstract

Liver diseases, including fatty liver, hepatitis, and cirrhosis, remain major global health challenges due to their disruption of metabolic homeostasis and detoxification processes. Redox imbalance plays a central role in liver disease progression by promoting inflammation, hepatic stellate cell activation, mitochondrial dysfunction, and fibrogenesis. Although flavonoids have historically been considered direct reactive oxygen species (ROS) scavengers, emerging evidence indicates that their biological effect at physiological concentrations are primarily mediated through modulation of intracellular redox signalling rather than simple radical neutralisation. This review highlights flavonoids as redox-modulating agents capable of restoring hepatic redox homeostasis through coordinated regulation of molecular pathways. Mechanistically, flavonoids activate the Nrf2-Keap1 axis to enhance endogenous antioxidant defences, including heme oxygenase-1 and glutathione biosynthesis enzyme, while suppressing NF-κB-mediated pro-inflammatory signalling and modulating MAPK and PI3K/Akt pathways. They also regulate mitochondrial redox balance, supporting mitophagy, metabolic adaptation, and cellular resilience to oxidative stress. In addition, flavonoid biotransformation by the gut microbiome improves intestinal barrier integrity, reduces endotoxin-driven hepatic inflammation, and contributes to gut–liver crosstalk. Collectively, these mechanisms position dietary flavonoids as multi-target redox modulators with promising therapeutic potential in chronic liver disease, although further studies are needed to improve their bioavailability and clinical translation.

## Simple Summary

Liver diseases, including fatty liver, hepatitis, and cirrhosis, are major global health concerns due to their impact on inflammation, detoxification, and metabolic imbalance. A key factor in their progression is oxidative stress. Dietary flavonoids, being naturally occurring compounds present in fruits, vegetables, and medicinal plants, have gained attention as they play a protective role in liver health. Flavonoids do not act merely as direct antioxidants but also influence cellular signalling pathways involved in inflammation, redox balance, and metabolism. They enhance the body’s own defence systems, suppress inflammatory signalling, and improve mitochondrial function and lipid metabolism. Additionally, they interact with gut microbiota, improving intestinal barrier integrity and reducing the transfer of harmful endotoxins to the liver, thereby strengthening the gut–liver axis. Experimental and clinical findings demonstrate that flavonoids can reduce liver injury, slow disease progression, and improve metabolic function. However, challenges such as variability in human responses and poor bioavailability highlight the need for further research. Overall, dietary flavonoids show promising multi-target agents for the prevention and management of chronic liver diseases.

## 1. Introduction

The liver is the largest internal organ of the body. It is indispensable for maintaining metabolic functions. It plays a central role in maintaining the metabolism of lipids, proteins, and carbohydrates, thereby supporting overall physiological balance. The liver detoxifies harmful endogenous and exogenous compounds, including drugs and toxins [1]. The organ also produces vital plasma proteins, including albumin and coagulation factors. It controls blood glucose regulation and produces bile needed for fat absorption and digestion [2]. The liver’s dual supply of blood is one of its distinctive features. It receives nutrient-rich blood from the portal vein and oxygenated blood from the hepatic artery. This unique vascular arrangement facilitates rapid exchange of nutrients and removal of toxic substances and xenobiotics from circulation [3]. Despite its remarkable regenerative and functional capacity, liver disease remains a major global health challenge. Annually, approximately millions of deaths are attributed to disorders, such as hepatocellular carcinoma, cirrhosis, and acute liver failure [4,5]. Viral and autoimmune hepatitis, alcoholic liver disease (ALD), non-alcoholic liver disease (NAFLD), and drug-induced liver injury are common conditions [6,7]. Among these, NAFLD has begun rising rapidly in prevalence due to an increase in obesity and metabolic syndrome rates [4]. Although existing therapies, including corticosteroids, antivirals, and immunosuppressants, can delay progression of disease, they are often associated with high costs, side effects, and decreased long-term efficacy [8]. In advanced cases, liver transplantation remains the last treatment option, but its use is considerably constrained by donor shortages and post-transplant complications [9]. Nonetheless, natural hepatoprotective compounds derived from medicinal plants are gaining significant interest from researchers [10]. Medicinal plants and plant-based supplementary diets are rich sources of polyphenolic compounds or flavonoids. Apples, berries, citrus fruits, grapes, onions, green tea, red wine, and cocoa are common dietary sources rich in flavonoids.

Medicinal plants, such as catechins from *Camellia sinensis*, baicalin from *Scutellaria baicalensis*, silymarin from *Silybum marianum*, curcumin from *Curcuma longa*, resveratrol from *Vitis vinifera*, ginkgetin from *Ginkgo biloba*, and rutin from *Sophora japonica*, are especially rich in bioactive flavonoids. They have exerted potent liver-protective effects by suppressing inflammation, lowering oxidative stress, and promoting tissue repair [11,12,13]. These characteristics position them as promising candidates for future therapeutic applications [14]. Flavonoids comprise a structurally diverse group of naturally occurring polyphenolic compounds abundantly found in grains, tea, fruits, vegetables, and numerous medicinal plants [15,16,17]. They serve as secondary metabolites and are responsible for UV protection, pigmentation, and defence mechanisms against pathogenic stress [18]. In humans, they are extensively studied for their wide spectrum of biological activities, including anti-inflammatory, antioxidant, antiviral, and anticancer effects [19]. Their ability to chelate metal ions neutralises reactive oxygen species (ROS) and regulates key cellular signalling pathways, particularly Nrf2 and NF-κB. This is central to maintaining cellular homeostasis and safeguarding hepatic tissue [20]. Recent studies confirm that flavonoids exert hepatoprotective effects against toxin-induced and metabolic disturbances by alleviating oxidative stress, inhibiting hepatocyte apoptosis, and improving lipid metabolism [21,22]. Thus, exploring the hepatoprotective potential of flavonoids in liver diseases offers a promising path for the advancement of effective plant-based hepatoprotective interventions. This review explores plant-derived flavonoids consumed as part of functional foods that support hepatic health primarily through their interactions with the gut microbiome. The purpose of this review is to consolidate current mechanistic evidence of flavonoid-mediated redox regulation in liver disease, with particular emphasis on intracellular signalling pathways and gut–liver interactions, and to assess their potential therapeutic relevance.

## 2. Chemistry and Classification of Flavonoids

Flavonoids represent an important class of plant-derived bioactive phytochemicals that are being explored for their potential to be used as medicine in liver disease. Their broad pharmacological profile, particularly antioxidant, anti-inflammatory, and metabolic regulatory activities, positions them as therapeutic candidates for both preventing and managing multiple hepatic disorders. With the global incidence of viral hepatitis, NAFLD, alcoholic liver disease, and drug-induced hepatotoxicity steadily rising, interest in the hepatoprotective value of flavonoids has intensified. Flavonoids are among the most diverse families of naturally occurring polyphenolic compounds in the plant kingdom. Their structures possess a 15-carbon skeleton, arranged in a C_6_–C_3_–C_6_ arrangement. It is composed of two aromatic rings (A and B) linked by three-carbon chains that form a heterocyclic pyran ring (C ring). Structural variation, such as differences in saturation of the C ring, oxidation level, and number or position of methoxy, hydroxyl, or glycosidic substituents, results in a wide range of compounds with distinct physicochemical and biological characteristics. These modifications significantly determine their solubility, cellular target binding, and antioxidant activity [16]. Flavonoid biosynthesis in plants takes place via the phenylpropanoid pathway. The process starts with the action of chalcone synthase, which catalyses the condensation of one molecule of *p-coumaroyl-CoA* with three molecules of *malonyl-CoA* to form chalcone. This intermediate subsequently undergoes cyclisation to yield the basic flavanone structure, which serves as a precursor for all other flavonoid subclasses [23]. The attachment of organic acids or sugars to the flavonoid core forms glycosides that enhance water solubility and influence its metabolic stability within biological systems.

### Subclasses of Flavonoids

**Figure 1:**
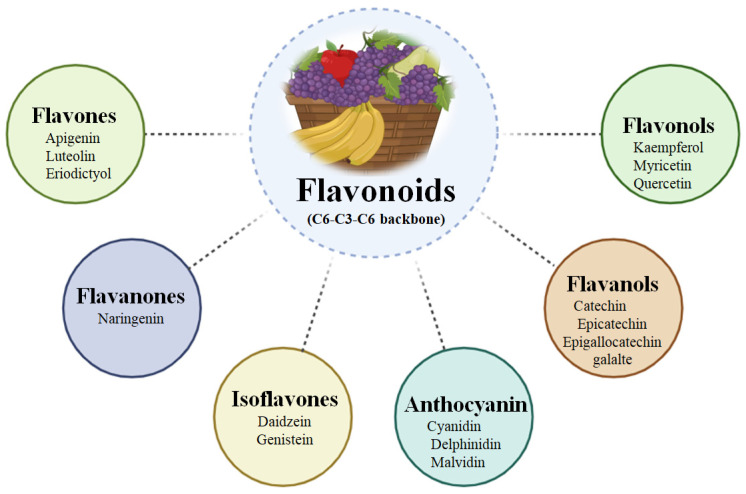
Major subclasses of dietary flavonoids.

Flavonoids are classified mainly on the degree of oxidation and the types of substitution pattern of the C ring. They share a basic C6–C3–C6 carbon backbone. They are categorised into several major subclasses (Figure 1), most notably flavones, flavonols, flavanols, anthocyanins, isoflavones, and flavanones [24,25,26,27]. Flavonoids share a common C6–C3–C6 backbone and are widely present in fruits and plant-derived foods. Based on structural variations, they are classified into several subclasses, including flavones (apigenin, luteolin, and eriodictyol), flavonols (kaempferol, myricetin, and quercetin), flavanols (catechin, epicatechin, and epigallocatechin gallate), flavanones (naringenin), isoflavones (daidzein, genistein), and anthocyanins (cyanidin, delphinidin, and malvidin).

Flavones such as apigenin and luteolin possess a double bond between carbons C_2_ and C_3_ and a ketone group at C_4_. They are abundant in high-concentration foods, such as celery, parsley, and chamomile [28,29]. They possess strong antioxidant and anti-inflammatory activities through modulation of Nrf2 and NF-κB pathways [30]. Flavonols such as quercetin, kaempferol, and myricetin differ by having a hydroxyl group located at the C_3_ position. These compounds are found abundantly in onions, apples, and tea [31,32]. They act as metal ion chelators, exhibit strong antioxidant capacity, and play a vital role in protecting liver cells from oxidative stress [1]. Flavanones, also known as dihydroflavones, such as naringenin and hesperidin, have a saturated C_2_–C_3_ bond, giving molecules a non-planar structure [33]. These are generally found in citrus fruits, such as oranges and grapefruits [34]. These compounds show notable lipid-modulating and anti-inflammatory properties, often associated with the AMPK pathway [35,36]. Isoflavones, including genistein and daidzein, possess a structurally unique feature in which the B ring is linked to the C_3_ position of the heterocyclic C ring. They are predominantly found in soybeans and other legumes [37]. A characteristic property is to exhibit oestrogen-like activity mediated through interaction with oestrogen receptors. They have shown potential in reducing oxidative damage and hepatic fibrosis [38]. Anthocyanidins are the aglycone (sugar-free) forms of anthocyanins and are responsible for the blue, red, and purple colouration of fruits like grapes and berries [39]. Their unique flavylium cation structure not only imparts colour but also confers strong antioxidant potential. It also contributes to hepatoprotective effects by inhibiting lipid peroxidation [40]. Flavanols are also known as catechins or flavan-3-ols. They lack both the carbonyl group at C_4_ and the double bond between C_2_ and C_3_. These are commonly found in cocoa, tea, and apples [41]. Catechins, particularly EGCG, exhibit potent antioxidants that support hepatic detoxification processes by suppressing inflammatory and oxidative stress [42,43].

**Figure 2:**
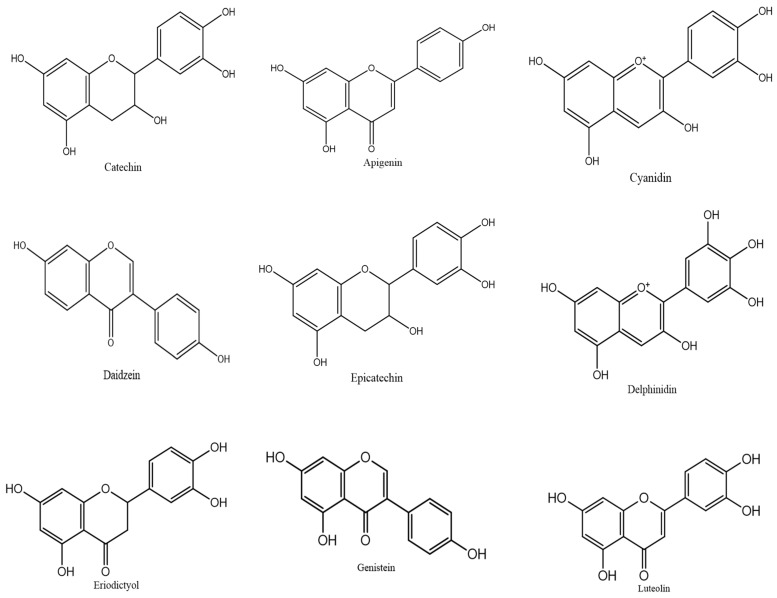

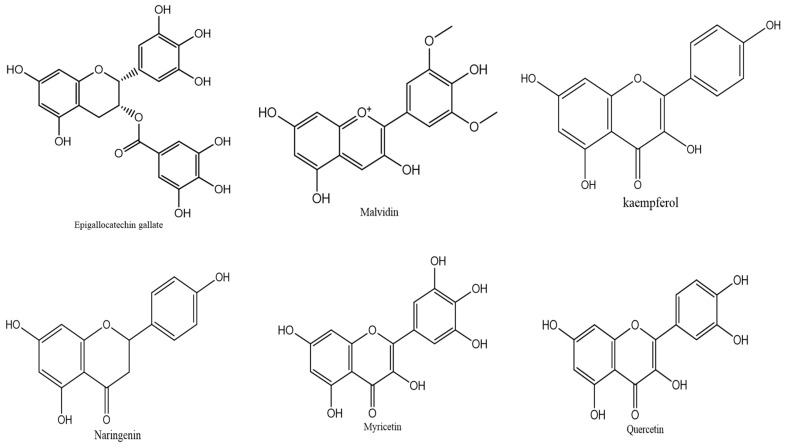
Chemical structures of common flavonoids.

Figure 2 depicts the structural diversity of major flavonoid classes, providing an overview of the chemical frameworks that underlie the biological activities of flavonoids in liver health.

## 3. Mechanisms of Hepatoprotection by Flavonoids

Table 1 summarises experimental and clinical observations on how different flavonoids confer liver protection via diverse cellular and molecular mechanisms. The evidence has been organised into major mechanistic categories. This includes antioxidant activity, anti-fibrotic effects, anti-inflammatory activity, mitochondrial protection, modulation of lipid metabolism, and regulation of hepatic signalling pathways. The complexity of flavonoid-mediated redox reprogramming in liver disease cannot be fully explained through simple pathway investigations. Multi-omics approaches offer a comprehensive view of flavonoids’ influence on liver health by simultaneously capturing alterations in the composition of gut microbiome, microbial and host metabolites, protein networks, and hepatic genes.

Metagenomic analysis reveals microbial taxa that respond to flavonoid intake, whereas lipidomic and metabolomic studies identify changes in short-chain fatty acids, bile acid profile, and phenolic metabolites linked to metabolic improvement and reduced steatosis. Transcriptomic, proteomic, and epigenomic analyses further highlight coordinated regulation of redox-sensitive and Nrf2-dependent antioxidant genes (HO-1, NQO1, and glutamate cysteine ligase), alongside modulation of inflammatory (NF-κB), MAPK, fibrotic, inflammatory oxidative stress, autophagy, mitochondrial biogenesis, and lipid metabolism pathways. This enables system-level integration of flavonoid microbiome liver interactions. Integration of these datasets through systems biology frameworks and network-based modelling enables identification of key signalling pathways, biomarker signatures, and predictive therapeutic targets. This supports precision nutrition strategies and strengthens the translational potential of flavonoid-mediated redox modulation in chronic liver diseases.

**Table 1:** Reports on mechanisms underlying hepatoprotective role of flavonoids.

Sr. No.	Mechanism	Flavonoid(s)	Main Findings	References
1.	Redox modulation and antioxidant	Kaempferol	Activates Nrf2 signalling, activates GPX4, lowers ROS; inhibits ferroptosis and APAP-induced liver injury.	[44]
		Kaempferol	Enhances AMPK/Nrf2 pathway, lowers MDA, and restores SOD/CAT/GPx activity.	[45]
		Quercetin	Demonstrates a consistent decrease in oxidative stress and antioxidant enhancement across fibrosis models.	[46]
2.	Modulation of anti-inflammatory markers (NF-κB, MAPK, cytokines)	Kaempferol	Inhibits NLRP3 inflammasome; reduces neutrophil-mediated hepatic inflammation.	[47]
		Kaempferol	Blocks the NF-κB, JNK, and MAPK pathways; reduces the hepatic inflammation load.	[36]
		Quercetin	Reduces M1 macrophage polarisation via Notch1 signalling; decreases pro-inflammatory cytokine release.	[45]
		Quercetin	Reduces inflammation-driven fibrosis via inhibiting NF-κB and p38 MAPK activation.	[5]
		Silymarin	Reduces NF-κB activation, cytokine release, and apoptosis in the NASH model.	[48]
3.	Anti-fibrotic effects (HSC inhibition, ECM remodelling, TGF-β)	Naringenin	Suppresses hepatic stellate cell activation and TGF-β signalling; strong anti-fibrotic action.	[49]
		Kaempferol	Attenuate fibrosis through miR-26b-5p/Jag1/Notch pathway, decreasing HSC activation.	[50]
		Quercetin	Decreases ECM accumulation by inhibiting HSC autophagy and blocking TGF-β1/Smad signalling.	[51]
		Quercetin	Limits collagen deposition and inflammation-related fibrosis via NF-κB/MAPK pathways.	[46]
4.	Metabolic regulation (steatosis, insulin resistance, lipid metabolism)	Multiple flavonoids	Improves ALT/AST, insulin levels, TGs, and BMI; supports systemic metabolic profile.	[52]
		Dietary flavonoids	Higher dietary intake is associated with lower NAFLD progression risk.	[53]
		Naringenin	Improves glucose tolerance, lipid levels, and reduces hepatic steatosis.	[54]
		Silymarin	Activates FXR, improves insulin sensitivity, and reduces steatosis in NASH models.	[55]
		Kaempferol	Modifies metabolic reprogramming in hepatic monocytes; supports metabolic homeostasis.	[56]
5.	Cell death pathways, ferroptosis, autophagy & gut–liver axis	Kaempferol	Prevents ferroptosis by activating Nrf2/GPX4 in APAP-induced liver injury.	[57]
		Quercetin	Inhibits autophagy in HSCs, reducing fibrosis progression.	[51]
		Kaempferol	Regulates apoptosis and immune–metabolic pathways in monocytes.	[56]
		Multiple flavonoids	Highlights flavonoid-mediated modulation of gut microbiota, endotoxin load, and gut–liver communication in MAFLD.	[58]

**Figure 3:**
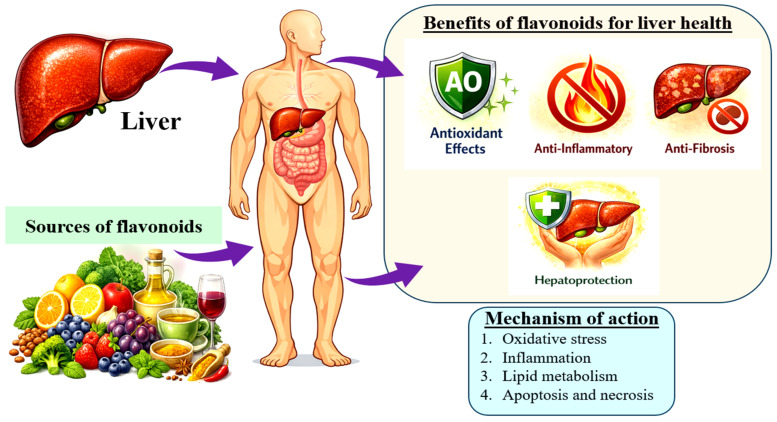
Beneficial effects of dietary flavonoids on liver health. Flavonoids derived from plant-based foods, such as fruits, vegetables, tea, and plant oils, contribute to liver health through multiple biological activities. These compounds exert antioxidant, anti-inflammatory, and anti-fibrotic effects, ultimately promoting hepatoprotection. The underlying mechanisms involve the regulation of oxidative stress, inflammatory pathways, lipid metabolism, and cell death processes, including apoptosis and necrosis.

Overall, flavonoids support liver health through multiple interconnected molecular pathways [59,60,61] (Figure 3):

1. **Antioxidant and redox regulation:** Flavonoids help to safeguard hepatic cells. They neutralise reactive oxygen species directly and also enhance endogenous antioxidant enzymes, such as catalase, superoxide dismutase, and glutathione. By limiting lipid peroxidation, they help protect hepatocytes in metabolic and toxin-induced liver injury models.
2. **NF-κB suppression and anti-inflammatory actions:** Flavonoids attenuate hepatic inflammation by downregulating signalling pathways, primarily NF-κB. It results in a reduction in the release of pro-inflammatory cytokines, including IL-1β, TNF-α, and IL-6. By moderating inflammatory cascades, flavonoids help limit immune-driven hepatocyte damage and may slow the progression from simple fatty liver to steatohepatitis.
3. **Anti-fibrotic activity:** In fibrosis models, flavonoids possess the ability to suppress hepatic stellate cell activation. They modulate TGF-β pathways and promote extracellular matrix remodelling. In preclinical studies, these effects are slow or partially reverse the progression of fibrogenesis.
4. **Metabolic regulation:** Flavonoids enhance insulin signalling, influence healthier lipid metabolism, and support mitochondrial function and biogenesis. Through these mechanisms, they improve features of MASLD/NAFLD and reduce hepatic fat accumulation and disturbances in energy homeostasis.
5. **Regulation of cell death and host defence:** They modulate apoptosis, autophagy, and ferroptosis pathways in both hepatocytes and stellate cells. Additionally, early research indicates some flavonoids also exhibit supportive antiviral properties, which further contribute to liver protection by reducing virus-induced tissue damage.

## 4. Redox Imbalance in Liver Pathophysiology

**Figure 4:**
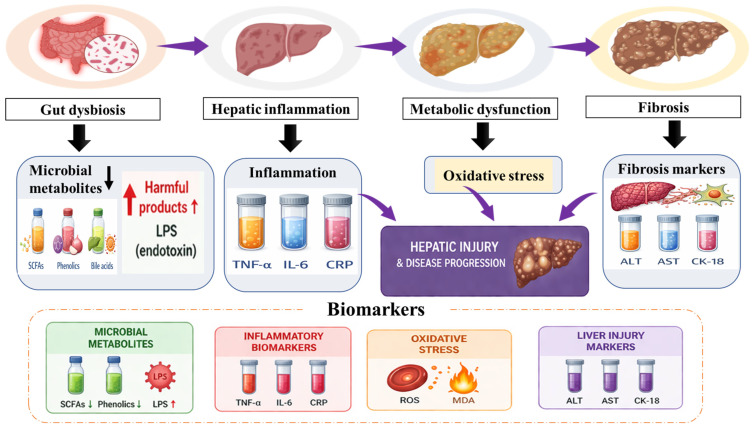
This schematic representation illustrates multifactorial mechanisms underlying the progression of liver disease through the gut–liver axis. Hepatic inflammation results from the combined influence of oxidative stress, lipid accumulation, immune activation, and gut dysbiosis. During gut dysbiosis, there is a reduction in beneficial microbial metabolites, including short-chain fatty acids (SCFAs) and certain phenolic compounds, along with an increase in harmful products such as lipopolysaccharides (LPS) and other pro-inflammatory mediators. This imbalance contributes to impaired intestinal barrier integrity and enhanced translocation of endotoxins to the liver. These changes, together with inflammatory mediators (TNF-α, IL-6, and CRP) and oxidative stress markers (ROS, MDA), drive hepatic injury. Clinical biomarkers (ALT, AST, and CK-18) reflect liver damage and disease progression.

The liver is essential for nutrient storage, central metabolism, and detoxification. Because of these properties, it is highly susceptible to damage caused by alcohol, toxic chemicals, and metabolic imbalances. Although liver disease arises from different causes, the majority of liver disorders involve common biological mechanisms, such as oxidative stress, hepatocyte loss, fat accumulation, inflammatory responses, and fibrosis. These pathways influence one another, leading to continuous liver damage and a gradual decline in liver function. In addition to these host-driven mechanisms, alterations in the gut microbiota (gut dysbiosis) further modulate disease progression through the gut–liver axis. Thus, hepatic inflammation should be viewed as a multifactorial process resulting from the combined influence of metabolic, immunological and microbial factors, as represented in Figure 4.

### 4.1. Oxidative Stress

It is one of the major contributors to liver damage. It generates when the reactive oxygen and nitrogen species surpass the liver’s antioxidant defence system, coordinated by enzymes such as CAT, SOD, and GPx. Excessive ROS levels injure cellular lipids, nucleic acids, and proteins, resulting in compromising hepatocyte function and integrity [21]. In disorders such as ALD, NAFLD, and drug-induced hepatotoxicity, mitochondria are a major intracellular source of ROS. In addition, cytochrome P450 enzymes, particularly CYP2E1, contribute to ROS generation during metabolic processing of xenobiotics [62]. Excessive lipid production leads to the formation of toxic lipid peroxidation products, such as 4-HNE and MDA, which bind to proteins and disrupt cell integrity. To mitigate oxidative stress, the body initiates an antioxidant response mainly regulated by Nrf2. Upon activation, Nrf2 translocates to the nucleus and induces the expression of cytoprotective enzymes like HO-1 and GCL. However, when this pathway is impaired, oxidative damage processes are unchecked, resulting in progressive hepatocyte dysfunction and cellular damage.

### 4.2. Inflammation

It plays a dual role in the progression of liver disease, functioning both as a trigger and an outcome of hepatic injury. Cellular damage within the liver leads to the generation of DAMPs, which stimulate Kupffer cells and recruit neutrophils and monocytes to the injury site [63]. Once activated, Kupffer cells secrete pro-inflammatory cytokines, including IL-1β, TNF-α, and IL-6, which enhance the hepatic inflammatory response. These cytokines activate pathways like NF-κB and MAPKs, causing the genes implicated in cell death and inflammation pathways. Sustained inflammatory signalling promotes the activation of hepatic stellate cells. This results in excessive ECM deposition and fibrosis progression. In immune-mediated conditions, such as cytotoxicity and viral hepatitis, further aggravate liver injury and inflammation. If the condition becomes chronic, it disrupts liver structure and increases the risk of malignant transformation, contributing to hepatocellular carcinoma [64].

### 4.3. Apoptosis and Necrosis

Numerous liver problems are characterised by hepatocyte death, which can be an uncontrolled process of necrosis or a controlled process of apoptosis. Apoptosis death involves signalling through death receptors like Fas and TNF receptors or through mitochondrial mechanisms mediated by Bcl-2 proteins and caspases [65]. Oxidative stress, ER stress, and inflammatory cytokines can activate caspase-3 and caspase-9, resulting in DNA breakage and cell shrinkage. Necrosis is distinguished by membrane rupture, cell swelling, and the uncontrolled leakage of intracellular components, which further amplifies inflammation by stimulating Kupffer cells and other immune cells. Acetaminophen toxicity is a classic example of necrotic liver injury, driven by the toxic metabolite NAPQI, which depletes glutathione and disrupts mitochondrial function [62]. Another related programmed pathway, necroptosis, also contributes to liver injury. This regulated form of necrosis is controlled by RIPK1, RIPK3, and MLKL, and is gaining interest as another contributor to liver pathology for protecting the liver.

### 4.4. Lipid Accumulation and Fibrosis

Lipid accumulation, also known as steatosis, is an early stage of liver disorders like ALD and NAFLD. It is caused by an imbalance between synthesis, oxidation, and absorption and lipid uptake. Triglycerides accumulate within hepatocytes, leading to mitochondrial dysfunction, lipotoxicity, and increased ROS production. Excessive accumulation of triglycerides within hepatocytes leads to lipotoxicity, mitochondrial dysfunction, and further ROS production. The c-Jun N-terminal kinase (JNK) and NF-κB pathways are activated by ensuring ER and oxidative stress, which promotes inflammation and apoptosis [66]. Fibrosis, which is the deposition of collagen and ECM proteins, is brought on by prolonged liver damage. The hepatic stellate cells (HSCs) are the primary event in fibrogenesis. HSCs are quiescent, but they transdifferentiate into myofibroblast-like cells that secrete collagen types I and III in response to transforming growth factor-beta (TGF-β) and platelet-derived growth factor (PDGF). Persistent matrix deposition ultimately leads to architectural distortion, cirrhosis, and impaired hepatic function.

### 4.5. Role of Gut Microbiota in Flavonoid Bioactivation and Liver Biomarkers

The gut microbiota plays a critical role in bio-transforming dietary flavonoids into smaller phenolic compounds and other metabolites with improved bioavailability and enhanced biological effects [67,68]. These strengthen antioxidant defences, regulate inflammatory pathways, and influence hepatic fluid and glucose metabolism. Nonetheless, variations in individual microbiomes influence flavonoid metabolism and may lead to therapeutic outcomes. Disruption of intestinal barrier function within the gut–liver axis facilitates the translocation of endotoxins, such as lipopolysaccharide (LPS), which induces inflammatory cascades and triggers hepatic oxidative stress. Flavonoids and their metabolites can improve intestinal barrier integrity, reduce LPS-mediated redox imbalance, and modulate bile acid signalling pathways, such as FXR and TGR5, that control metabolic and inflammatory processes. It is important to note that flavonoids do not act as direct ligands for FXR and TGR5 but instead modulate these signalling pathways indirectly through alterations in gut microbiota composition and bile acid metabolism. Furthermore, microbial-derived products, including short-chain fatty acids and secondary bile acids, modulate redox-sensitive hepatic pathways [69,70]. Collectively, these influences gut–liver communication and support redox homeostasis in chronic liver disease.

Flavonoid biotransformation by the gut microbiota plays a crucial role in determining their bioavailability and biological activity. Most dietary flavonoids are present in glycosylated forms, which are poorly absorbed in the small intestine and therefore reach the colon, where they undergo extensive microbial metabolism. Gut bacteria enzymatically convert these compounds through processes such as deglycosylation, dehydroxylation, demethylation, and ring fission, resulting in the formation of smaller phenolic metabolites with enhanced bioavailability and biological activity [71,72]. These metabolites exhibit potent antioxidant and anti-inflammatory properties and contribute to maintaining intestinal barrier integrity by strengthening tight junctions and reducing epithelial permeability [73]. In addition, microbial metabolites of flavonoids have been shown to suppress endotoxin-induced inflammation by modulating key signalling pathways, including NF-κB, thereby reducing lipopolysaccharide (LPS)-mediated hepatic injury [74]. Furthermore, these interactions support bidirectional communication within the gut–liver axis, highlighting the importance of gut microbiota in mediating the therapeutic effects of flavonoids [70,72].

Recent studies highlight the central role of the gut–liver axis in the progression and management of liver diseases. Modulation of gut microbiota and its metabolites has emerged as a critical mechanism influencing hepatic inflammation and fibrosis. For instance, *Auricularia auricula* polysaccharides were shown to attenuate hepatic fibrosis by enhancing short-chain fatty acid (SCFA) production and regulating gut microbial composition, thereby reducing inflammatory responses [69]. Similarly, alterations in the gut microbiome have been linked to ferroptosis-mediated liver injury in metabolic dysfunction-associated fatty liver disease (MAFLD), suggesting a novel mechanistic pathway [75]. Broader reviews further emphasise that gut dysbiosis contributes to liver disease through immune modulation, endotoxemia, and metabolic disturbances, reinforcing the clinical significance of the gut–liver axis [67,68]. Collectively, these findings underscore the therapeutic potential of targeting gut microbiota and microbial metabolites in liver disease management.

Liver damage is typically evaluated through serum biomarkers that indicate hepatic function and hepatocyte integrity. Alanine aminotransferase (ALT) and aspartate aminotransferase (AST) are primary indicators of hepatocellular damage. ALT is more liver-specific, and AST can also rise in muscle damage. Alkaline phosphatase (ALP) levels typically rise in cases of bile duct obstruction and cholestatic disorders. Gamma-glutamyl transferase (GGT) serves as a marker of biliary injury and is also associated with oxidative stress. Elevated total bilirubin levels indicate impaired bilirubin excretion or metabolism and are clinically associated with jaundice. Additionally, they are associated with reduced serum albumin and prolonged prothrombin time (PT). Both indicate the liver’s ability to synthesise proteins. These are microbial-derived and complement traditional clinical markers such as ALT, AST, and CK-18, offering a more integrated understanding of liver dysfunction within the context of the gut–liver axis.

In addition, tools such as CK-18 and specific microRNAs (miR-122 and miR-192), and extracellular vesicles, are becoming important tools for earlier detection and for distinguishing among different types of liver disease [76]. Liver disorders, including NASH, MASLD, ALD, and fibrosis, represent a global health burden that extends beyond clinical settings to encompass environmental exposures, dietary patterns, and microbiome disruptions. The limited effectiveness of drug-focused therapies addressing these complex, multifactorial conditions highlights the need for integrative approaches that link gut microbiota, diet, and environmental factors. Moving beyond the traditional antioxidant paradigm, this perspective reframes flavonoids within system-based nutrition and omic-driven functional foods, positioning liver disease within one health challenge rooted in sustainable food systems. The One Health perspective underscores interactions among agricultural systems, diet, environmental factors, and antibiotic use in shaping the gut microbiome in both humans and animals. It collectively influences liver diseases. Within this context, the gut–liver axis functions as a key integrative signalling hub, where portal circulation, microbial metabolites, and bile acids transmit the effects of metabolic disturbances across species.

## 5. Clinical Evidence and Limitations

### 5.1. Clinical Evidence in Humans and Specific Liver Disorders

Clinical and translational findings provide strong support for flavonoid-rich extracts in liver care. Studies on silymarin, a milk thistle extract, show that it can lower ALT and AST levels in CCl_4_-induced liver injury and reduce the expression of inflammatory genes [77,78,79]. In addition, a niosomal combination of quercetin and silymarin enhanced antioxidant defences and helped restore normal liver structure in a rat liver-cancer model, indicating promising relevance for future human applications [80].

This explores the flavonoid actions in major liver disorders through a microbiome-focused perspective rather than a broad, nonspecific hepatoprotective approach. In MASLD/NAFLD and NASH, increased endotoxin contributes to metabolic inflammation and insulin resistance. Flavonoids contribute to restoring microbial balance, lowering endotoxemia, and improving hepatic lipid and glucose homeostasis [67]. During liver fibrosis and cirrhosis, impaired intestinal barrier function and release of microbe-derived profibrotic signals promote hepatic stellate cell activation. Flavonoids counter these processes by reinforcing gut integrity and suppressing fibrogenic signalling pathways [81,82]. In ALD and cholestatic liver disorders, alcohol-related microbial alterations and disrupted bile acid dysregulation exacerbate liver injury. Flavonoids modulate ethnomicrobiome interactions and regulate bile acid FXR signalling to support hepatic detoxification and metabolic control [67,73].

### 5.2. In Vitro and In Vivo Studies

Flavonoids like quercetin, kaempferol, apigenin, and luteolin demonstrate high liver protection in cell-based models. They reduce oxidative stress and inflammatory reactions in primary hepatocytes and HepG2 cells. Quercetin-7-rhamnoside raised glutathione, reduced ROS production, and enhanced antioxidant enzymes in H_2_O_2_-challenged L-02 cells. It also protected mice from CCl_4_ toxicity by lowering ALT, AST, and lipid peroxidation [83]. Kaempferol provided similar benefits in acetaminophen-injured hepatocytes by decreasing inflammatory mediators, maintaining mitochondrial integrity, and activating SIRT1 signalling [84].

Animal studies using CCl_4_, acetaminophen, and high-fat diet models consistently demonstrate the liver-protective actions of flavonoids. In CCl_4_-induced injury, a combined silymarin–quercetin system markedly restored antioxidant enzymes, reduced ALT, AST, and hepatic lipid buildup, and improved overall liver morphology [85]. In rats exposed to acetaminophen, kaempferol lowered oxidative stress, activated SIRT1, suppressed CYP2E1, and decreased caspase-3 activity along with pro-inflammatory cytokines, such as TNF-α and IL-6 [84]. In high-fat diet-driven fatty liver (MASLD), quercetin together with silibinin reduced fat accumulation and inflammation by modulating lipid-handling proteins, including CD36 and PLIN3 [86].

Recent studies have further strengthened the evidence supporting the hepatoprotective effects of plant-derived flavonoids. For example, quercetin and kaempferol have demonstrated significant antioxidant and anti-inflammatory activities in experimental models of non-alcoholic fatty liver disease (NAFLD) and liver fibrosis, primarily through modulation of Nrf2 and NF-κB signalling pathways. Naringenin has been shown to improve hepatic lipid metabolism and reduce steatosis in high-fat diet-induced models, while epigallocatechin gallate (EGCG), a major catechin from green tea, has been reported to attenuate oxidative stress and inflammatory responses in both in vitro and in vivo studies. In addition, emerging research highlights the ability of these flavonoids to influence gut microbiota composition, enhance intestinal barrier integrity, and reduce endotoxin-mediated hepatic injury, thereby reinforcing their role in the gut–liver axis. These recent findings further validate the therapeutic potential of flavonoids in liver disease management.

Mechanistically, gut dysbiosis contributes to liver disease through multiple interconnected pathways involving immune, metabolic, and barrier dysfunction. Disruption of intestinal barrier integrity increases gut permeability, allowing translocation of microbial products such as lipopolysaccharide (LPS) into the portal circulation, which activates Toll-like receptor 4 (TLR4) on hepatic Kupffer cells and triggers NF-κB-mediated inflammatory responses [67,68]. Concurrently, alterations in gut microbiota composition disrupt bile acid metabolism, leading to dysregulation of key signalling pathways mediated by farnesoid X receptor (FXR) and Takeda G protein-coupled receptor 5 (TGR5), thereby affecting hepatic metabolic and inflammatory homeostasis. Reduced production of beneficial microbial metabolites such as short-chain fatty acids (SCFAs) further impairs intestinal barrier function and anti-inflammatory signalling, exacerbating liver injury. Despite these advances, current understanding remains limited, as much of the evidence is derived from preclinical models with variable translational relevance to humans. In addition, inter-individual variability in gut microbiota composition, dietary influences, and methodological differences across studies complicate the interpretation of findings. Emerging evidence also suggests complex interactions between the gut microbiome, host metabolism, and ferroptosis-related pathways, although these mechanisms require further clarification [75]. Collectively, while the gut–liver axis represents a critical pathway in liver disease pathogenesis, further well-designed clinical and mechanistic studies are needed to fully elucidate these interactions and validate therapeutic strategies.

### 5.3. Specific Flavonoids and Their Hepatoprotective Actions

A wide range of flavonoids, most notably quercetin, silymarin, naringenin, hesperidins, luteolin, apigenin, epigallocatechin gallate (EGCG), and genistein, have been explored for their protective effects on the liver, supported by strong antioxidant, anti-inflammatory, anti-fibrotic, and metabolic actions. Quercetin acts largely by scavenging ROS, enhancing Nrf2-driven antioxidant defences, and suppressing NF-κB-mediated cytokine release, with studies showing reduced enzyme markers of liver injury and improved redox balance in toxin-induced models, as well as benefits in NAFLD through AMPK activation. Silymarin, rich in silybin, offers one of the most clinically validated hepatoprotective profiles, functioning through Nrf2 activation, NF-κB and TGF-β/Smad inhibition, membrane stabilisation, and stimulation of hepatocyte regeneration, with consistent improvements observed in both animal studies and clinical trials for chronic liver disorders. Naringenin and hesperidins, two citrus flavanones, support liver health by modulating lipid metabolism via AMPK and PPARα, reducing inflammation, and restoring antioxidant defences, showing particular promise in fatty liver, metabolic syndrome, and chemically induced injuries. Luteolin and apigenin are the two major occurring dietary flavones that contribute to liver protection by modulating signalling routes, such as MAPKs, PI3K/Akt, NF-κB, and Nrf2. Through these, they help limit oxidative stress, suppress inflammatory mediators, and attenuate fibrosis by inhibiting HSC activation. EGCG, a major catechin in green tea, exhibits potent anti-fibrotic and antioxidant effects by suppression of CYP2E1-mediated ROS, improving lipid metabolism, and also prevents hepatocyte apoptosis. Early clinical findings support its use in NAFLD, regulation of lipid metabolism, and protection against apoptosis, with emerging human evidence supporting its use in NAFLD, albeit with caution at high doses. Genistein, an important soy-derived isoflavone, protects liver health by acting on oestrogen receptors and metabolic pathways, better lipid regulation, lower fibrotic activity, and reduced inflammation, making it particularly relevant for oestrogen-deficient or metabolic liver conditions. Collectively, these flavonoids represent potential natural therapeutic agents for various liver disorders through their synergistic and multi-targeted mechanisms of action. Table 2 summarises the role of major flavonoids in liver disease based on common experimental evidence.

Accumulating evidence highlights the significant hepatoprotective potential of plant-derived flavonoids through multiple molecular mechanisms. Oxidative stress has been identified as a central driver of liver disease progression, and antioxidants such as flavonoids play a crucial role in mitigating hepatic injury [82]. Recent studies demonstrate that kaempferol attenuates liver fibrosis by suppressing macrophage M1 polarisation and inhibiting MAPK/NF-κB signalling pathways [81], while quercetin exerts anti-inflammatory and metabolic regulatory effects by modulating key markers such as VCAM-1 and enzymes involved in glucose and lipid metabolism [80]. Similarly, citrus flavanones such as naringenin and naringin have been shown to improve lipid metabolism and reduce steatosis in NAFLD models [87,88]. Green tea-derived polyphenols, particularly epigallocatechin-3-gallate (EGCG), have also demonstrated protective effects against liver injury by reducing oxidative stress, inflammation, and metabolic dysfunction, with both experimental and dietary studies supporting their role in liver health [89,90]. Collectively, these findings reinforce the therapeutic potential of flavonoids as multi-target agents in the prevention and management of liver diseases.

**Table 2:** Role of flavonoids in liver disease management.

Sr. No.	Flavonoid(s)	Design/Model	Findings	References
1.	Mixed flavonoids	Narrative/systematic review	Highlights antioxidant, anti-inflammatory, metabolic, and anti-apoptotic pathways as common mechanisms underlying hepatoprotection.	[91]
2.	Quercetin	Randomised clinical trial (NAFLD patients)	12-week quercetin treatment led to a decrease in liver fat and improved metabolic parameters when compared with the placebo group.	[92]
3.	Silymarin (silibinin complex)	Systematic review/meta-analysis	Silymarin can lower ALT and AST levels in clinical trials; results vary due to heterogeneity in formulation and outcome measures across trials.	[93]
4.	Naringenin	Rodent of CCl_4_ fibrosis model	Naringenin reduced inflammation, tissue necrosis, and fibrotic changes; the mechanism included antioxidation and NF-κB inhibition.	[49]
5.	Various flavonoids	Systematic review + meta-analysis	Flavonoids improved the liver enzymes and some histological or biomarker measures. Though large, well-designed RCTs are needed.	[52]
6.	Total flavonoids, flavanones, flavan-3-ols, etc.	Prospective cohort (humans)	Individuals with regular dietary flavonoid intake exhibited a lower risk of NAFLD progression, mediated by reductions in cholesterol and insulin resistance.	[53]
7.	Naringenin	Review/mechanistic	Naringenin reduced oxidative stress, suppressed the TGF-β pathway, blocked hepatic stellate cell activation, and regulated lipid metabolism and inflammation.	[49]
8.	Naringenin	Systematic review	Naringenin maintained the energy balance, improved lipid/glucose metabolism, and reduced inflammation and oxidative stress in NAFLD models.	[54]
9.	Flavonoids (various)	Review + clinical/preclinical	Flavonoids modulate gut microbiota, reduce endotoxemia, activate AMPK/Nrf2, suppress NF-κB, and improve liver fat in MAFLD.	[58]
10.	Kaempferol	Rat model	Kaempferol reduced inflammation (TNF-α, IL-1β), inhibited NF-κB and MAPK signalling, and activated AMPK/Nrf2 to reduce oxidative damage.	[38]
11.	Kaempferol	Mouse + cell line	Kaempferol activated Nrf2, increased GPX4, and reduced ROS and iron overload, preventing ferroptosis and liver injury after acetaminophen.	[44]
12.	Kaempferol	Mouse fibrosis + HSC in vitro	Kaempferol suppressed HSC activation by upregulating miR-26b-5p, downregulating Jag1, and inhibiting Notch signalling.	[50]
13.	Kaempferol	Mouse NASH + cell model	Kaempferol reduced inflammasome (NLRP3) activation, neutrophil-mediated inflammation, and lipid accumulation.	[47]
14.	Kaempferol	Mouse fibrosis + omics	Kaempferol improved fibrosis markers, altered gut microbiota composition, and reprogrammed monocytes via metabolic/transcriptomic changes.	[56]
15.	Quercetin	Mouse (BDL and CCl_4_ fibrosis)	Quercetin reduced fibrosis, downregulated ECM via TGF-β/Smad, and inhibited excessive autophagy via PI3K/Akt.	[51]
16.	Quercetin	CCl_4_ model + macrophage in vitro	Quercetin reduced macrophage infiltration and inhibited M1 polarisation (via Notch1), lowering inflammatory cytokines and fibrosis.	[94]
17.	Quercetin	CCl_4_-treated rats	Quercetin reduced NF-κB activation and p38 MAPK phosphorylation, modulated apoptosis (Bax/Bcl-2), and lowered fibrosis markers.	[95]
18.	Quercetin	Meta-analysis of animal studies	Quercetin improved biochemical ALT/AST, fibrosis markers (HA, LN) in fibrotic animals; mechanisms: anti-inflammatory, anti-oxidant, TGF-β, AMPK pathways.	[46]
19.	Silymarin/Silibinin	Mouse model of NASH	Silymarin reduced apoptosis, inflammation, and fibrosis; inhibited NF-κB, activated FXR, and improved metabolic parameters.	[48]

### 5.4. Bioavailability and Pharmacokinetics of Flavonoids

Despite flavonoids showing strong hepatoprotective effects in experimental models, the clinical trials of flavonoids remain limited due to poor bioavailability and rapid metabolic breakdown in the body. Flavonoids mainly occur as glycosides, which are 1st hydrolyzed in the gastrointestinal tract to form aglycones. Once they get absorbed through passive diffusion in the small intestine or transporter-mediated mechanisms, they undergo significant phase II metabolism. This generates sulphate, methylated and glucuronide metabolites that circulate bound to plasma proteins and reach organs, such as the liver, before being subsequently excreted. The effect on systemic availability is influenced by food components, first-pass metabolic processes, glycosylation patterns, and activity of efflux transporters like P-glycoprotein and multidrug resistance proteins. To address these limitations, advanced delivery systems such as liposomes, phytosomes, polymeric nanoparticles, solid lipid nanoparticles, nanoemulsions, and cyclodextrin complexes have been developed to improve stability, solubility, intestinal permeability, and hepatic targeting [96,97,98,99,100,101]. These technologies marked improvements in bioavailability and pharmacokinetic characteristics like area under the curve, elimination half-life, and hepatic accumulation. By preserving their biological activity, reducing dose requirements, and enhancing consistency in clinical hepatoprotective outcomes, the technical advancements eventually increase the translational potential of flavonoid-based interventions. Dietary flavonoids act as functional foods that support liver health with major classes derived from diverse plant sources. They are strongly influenced by farm-level factors such as crop variety, soil quality, and agricultural practices. Despite limited direct absorption, flavonoids undergo extensive gut microbial transformation, generating bioactive metabolites that exert hepatoprotective effects [102,103,104,105].

### 5.5. Safety, Toxicity, and Drug Interactions

When consumed through diet, flavonoids are usually considered safe. But their safety profile becomes dose-dependent when they are utilised in concentrated form in therapeutic formulations. They promote anti-inflammatory, antioxidant, and cytoprotective activity at the dietary level. In contrast, some flavonoids have adverse effects, including pro-oxidant stress, mitochondrial dysfunction, or cytotoxicity at higher pharmacological doses. These effects have been generally documented in compounds like EGCG and quercetin when consumed in excessive amounts, where high doses have been associated with hepatotoxicity and pro-oxidant effects [106,107,108,109]. However, many flavonoids, especially silymarin and naringenin, demonstrated safety profiles during prolonged use. Mostly, high doses of isoflavones can increase strain or alter endocrine balance in liver detoxification pathways. Most indicate low acute toxicity based on high LD50 values, although complete data remain inadequate for long-term duration, reproductive, and developmental safety. Furthermore, flavonoids may interact with conventional antiviral and hepatoprotective drugs. Various flavonoids affect phase-II metabolism, CYP enzymes, and transporters like P-gp, which alter the efficacy and bioavailability of drugs. Therefore, co-administration requires a careful pharmacokinetic assessment to maximise therapeutic advantages while minimising unfavourable herb–drug interactions [110,111,112,113].

## 6. Applications

Flavonoid research has evolved far beyond early experimental studies. Both dietary and pharmacological approaches demonstrate practical value in restoring liver health. Their main practical application areas include MASLD/NAFLD management, nutraceutical applications, anti-fibrotic interventions, and the development of advanced drug delivery systems.

### 6.1. Dietary and Nutraceutical Applications

Consuming foods rich in flavonoids, such as citrus fruits, berries, green tea, and various vegetables, can help to strengthen the liver’s defence system. In addition to dietary consumption, standardised supplements like silymarin obtained from the milk thistle plant or quercetin are already utilised in some clinical settings. These approaches serve as safe, accessible ways to enhance hepatoprotection, mainly through antioxidant and anti-inflammatory actions.

Flavonoid-rich medicinal plants, including species of the genus Artemisia [114], provide dietary phytochemicals that interact with the gut microbiome to influence liver health [115,116]. These are poorly absorbed by their own but undergo extensive microbial conversion into more bioavailable and bioactive metabolites, which underscores the key role of microbiome in flavonoid functionality. Microbial metabolism of flavonoids has been shown to alter microbial composition and function. It, in turn, can affect intestinal barrier function and immune responses that are central to the gut–liver axis and pathogenesis of liver disorders such as NAFLD and fibrosis. Experimental studies show that extracts from *Artemisia argyi* can improve gut microbial balance and reduce liver fibrosis in experimental models by regulating targeted microbes and hepatic signalling pathways [117]. Liver health is influenced by flavonoids through interconnected stages, ranging from cultivation and food processing to human dietary habits and metabolism. Sustainable farming supports soil health, and plant-associated microbiomes enhance flavonoid accumulation, while food processing, cooking practices, and post-harvest handling influence their stability and microbial metabolism. Regular consumption of minimal processes and a plant-based diet promotes gut microbial conversion of flavonoids into metabolites that regulate lipid metabolism, hepatic inflammation, and fibrosis. Together, this shows that sustainable farming and food systems help to prevent liver disease, which also supports environmental sustainability and improves public health.

### 6.2. MASLD/NAFLD Management and Fibrosis Prevention

Clinical evidence shows that certain flavonoids can reduce liver enzymes and decrease liver fat deposition, especially when combined with a healthy diet and exercise. Even so, flavonoids currently function as supportive therapies rather than independent treatments for fatty liver disease [117,118]. Extensive preclinical studies show that flavonoids regulate TGF-β-driven pathways, suppress hepatic stellate cell activation, and regulate extracellular matrix dynamics. These actions slow or, in some cases, partially reverse fibrosis in animal studies. The successful translation of these benefits to clinical practice will require large, well-designed clinical trials specifically focused on fibrosis-related outcomes [119,120].

### 6.3. Synergistic Role

To enhance flavonoid functionality, probiotics, fermented foods, and symbiotics act synergistically by improving microbial populations and facilitating their enzymatic transformations into more active metabolites. Fermentation further increases flavonoid bio-accessibility, with traditional fermented foods providing culturally appropriate delivery matrices that promote effective gut–liver interactions. This approach emphasises the food matrix in determining flavonoid efficacy, extending beyond the effects of isolated bioactive compounds [121,122,123].

Several studies have demonstrated that flavonoids modulate the gut–liver axis through their effects on specific microbial metabolites. For instance, quercetin has been shown to enhance the production of short-chain fatty acids (SCFAs), particularly butyrate, which improves intestinal barrier integrity and reduces systemic inflammation. Similarly, naringenin and its glycoside naringin have been reported to regulate bile acid metabolism and improve lipid homeostasis, thereby attenuating hepatic steatosis [87,88]. Epigallocatechin gallate (EGCG), a major polyphenol in green tea, has been shown to reduce circulating levels of lipopolysaccharide (LPS) by modulating gut microbiota composition and improving gut barrier function, ultimately decreasing endotoxin-driven hepatic inflammation [68,90]. Additionally, flavonoid-induced shifts in microbial metabolism led to the generation of bioactive phenolic metabolites, which exhibit antioxidant and anti-inflammatory effects within the liver. Collectively, these findings highlight how flavonoids influence key metabolites such as SCFAs, bile acids, and LPS, thereby playing a crucial role in regulating gut–liver axis signalling and liver health.

### 6.4. Translational and Clinical Applications

Advances in gut microbiome research are enabling the development of personalised nutrition strategies based on individual microbial profiles [124]. Such strategies allow flavonoid-rich diets or functional foods to be tailored according to gut microbial composition, thereby improving liver health and outcomes in metabolic liver disorders. Evidence from precision dietary interventions indicates that a special microbial profile can inform dietary recommendations and enhance production in metabolic liver disease by targeting gut microbiome taxa and metabolites associated with disease phenotypes [125,126,127]. The integration of multi-omics data, including metabolomics, metagenomics, and microbiome profiling, further enables the identification of microbial patterns linked to the progression of liver disorders and responses to flavonoid intake [128,129]. It supports the rational design for precision functional foods that optimise gut–liver interactions [130]. Such microbiome-guided dietary modulation may reduce dependence on conventional pharmacotherapy, lowering the healthcare costs and treatment burden [131]. Collectively, microbiome-informed flavonoid interventions represent an emerging frontier in precision hepatology where microbial ecology, diet, and host metabolism converge to guide preventive and targeted liver care.

### 6.5. Advanced Delivery Technologies

Innovative formulation approaches, such as liposomal systems and nano or polymer-based nanoparticles, are improving the clinical potential of flavonoids by improving their bioavailability and facilitating targeted delivery efficiently to hepatic tissue. These advanced delivery systems enable therapeutic efficacy at lower doses, thereby increasing the therapeutic use of compounds such as silybin and quercetin. A broad review of flavonoids highlights their potential in NAFLD, summarising antioxidant, anti-inflammatory, and metabolic regulatory effects, as well as their influence on the gut–liver axis [132]. Survey of flavonoid sources, structures, and their hepatoprotective effects in liver injury and toxicity [91]. Focused review on how quercetin may ameliorate NAFLD via its anti-inflammatory, antioxidant, metabolic, and anti-apoptotic actions [85]. Integrates animal/cell studies and gene-expression analyses to show how quercetin modulates lipid metabolism, bile acid pathways, inflammation, and gut microbiota in NAFLD [133]. Comprehensive mechanistic review showing how kaempferol protects against steatosis, inflammation, and fibrosis via SIRT1/AMPK, TGF-β/Smad, NF-κB, and by modulating the gut microbiota [134]. Clinical RCT showing that quercetin supplementation (500 mg) for 12 weeks reduced liver fat (measured by MRI) in patients with NAFLD [94]. An animal study showing quercetin promotes autophagy (via Beclin-1, LC3, p62) in NAFLD, contributing to lipid clearance and reduced steatohepatitis [135]. Mechanistic review on how flavonoids counteract xenobiotic (benzo[a]pyrene) liver injury via antioxidant pathways, phase I/II metabolism, and detoxification [136,137]. Preclinical study showing that fruit-derived flavonoid mixtures protect against alcoholic liver injury by activating Nrf2, inhibiting ferroptosis, and modulating gut microbiota [138]. A meta-analysis of RCTs in NAFLD shows that flavonoid supplementation improves liver enzymes, lipid profile, and inflammatory markers.

Dietary plant-based flavonoids have demonstrated significant therapeutic potential across a range of clinical and experimental settings. Evidence from randomised controlled trials, cohort studies, and intervention studies highlights their role in improving cardiometabolic health, glycaemic control, cognitive function, and inflammatory conditions. To provide a concise overview of these applications, key experimental and clinical studies are summarised in Table 3.

**Table 3:** Summary of representative experimental and clinical studies on dietary plant-derived flavonoids and their biological effects.

Sr. No.	Flavonoid Source	Flavonoid Class	Major Compounds	Study Type	Experimental Model	Main Biological Effects	References
1.	Green tea	Flavan-3-ol	Epigallocatechin gallate (EGCG) with piperine as bioenhancer	Experimental study	*Dugesia tigrina* (planarian regeneration mode)	Dose-dependent effects on tissue regeneration, modulate regeneration rate, suggest influence on oxidative stress pathways and on cell proliferation; potential enhancement with piperine (bioavailability effect)	[139]
2.	Soybean	Isoflavone	Genistein	In vitro	Cancer cell lines	Downregulation of miR-155 → anticancer activity	[140]
3.	Tea	Flavanol	EGCG	Clinical trial	Healthy human volunteers	Improved bioavailability and antioxidant status	[141]
4.	Tea extract	Flavanol	EGCG	Clinical trial (RCT)	Obese type 2 diabetic patients	Improved insulin resistance	[142]
5.	Fruits & vegetables	Flavonol	Quercetin	In vivo	Obese mice	Reduced inflammation via AMPKα1/SIRT1 pathway	[143]
6.	Hawthorn species (*Crataegus azarolus*, *Crataegus monogyna*) twigs	Flavonols and Flavan-3-ols	Quercetin, Kaempferol, Catechin, Epicatechin (along with other polyphenols)	Experimental study	In vitro biochemical assays (antioxidant and antimicrobial activity tests); metabolomic profiling (analytical chemistry techniques such as LC-MS)	Strong antioxidant activity (free radical scavenging); moderate antimicrobial activity; rich polyphenolic profile contributing to potential therapeutic effects	[144]
7.	Fruits & vegetables	Flavonol	Quercetin	In vivo	Obese Zucker rats	Improved metabolic syndrome and inflammatory status	[145]
8.	Flavonoid-rich diet	Mixed flavonoids	Multiple (quercetin, catechins, Anthocyanins)	Cohort study	Humans (UK Biobank)	Lower risk of type 2 diabetes	[146]
9.	Grape and blueberry extract	Anthocyanins/flavonols	Anthocyanins/flavan-3-ols	Randomised, double blind, placebo-controlled clinical trial	Older adults with mild cognitive impairment	Improved cognitive function	[147]
10.	Fruits (berries, apples, grapes), vegetables (onion, leafy greens), tea, cocoa	Mixed flavonoids (flavonols, flavan-3-ols, flavones, anthocyanins)	Quercetin, kaempferol, myricetin, catechin, epicatechin, EGCG, luteolin	Integrative study (network pharmacology + experimental + epidemiological evidence)	In silico (network pharmacology, target prediction, pathway analysis), supported by in vitro experiments and epidemiological datasets	Demonstrates polypharmacological (multi-target) effects; antioxidant and anti-inflammatory actions; modulation of key signalling pathways (PI3K/Akt, MAPK, NF-κB); identifies multiple protein targets and biological networks; supports role in chronic disease prevention	[148]
11.	Polyphenol-rich nutraceuticals	Mixed flavonoids	Multiple (anthocyanins, flavonols)	Randomised, double blind, placebo-controlled clinical trial	Human subjects	Improved cognitive function and neuroprotective biomarkers	[149]
12.	Strawberry	Anthocyanins/flavonoids	Pelargonidin, ellagic acid derivatives	Randomised controlled crossover trial	Adults with metabolic syndrome	Improved cardiometabolic risk markers	[150]
13.	Raspberry leaf tea	Flavonoids (polyphenols)	Flavonols, tannins	Clinical study	Healthy adults	Reduced postprandial glucose and insulin response	[151]
14.	Dietary anthocyanins (fruits/berries)	Anthocyanins	Cyanidin, delphinidin	Observational (population-based study)	US adult population	Lower risk of non-alcoholic fatty liver disease	[152]
15.	Dietary flavonoid intake	Mixed flavonoids	Quercetin, catechins, anthocyanins	Observational (UK Biobank study)	Human population	Lower risk of non-alcoholic fatty liver disease	[153]
16.	Green tea beverage	Flavanols	EGCG, catechins	Clinical study	Dyslipidemic human subjects	Improved cardiometabolic risk biomarkers	[154]
17.	Dietary polyphenol supplementation	Mixed flavonoids	Multiple	Randomised controlled trial	Adult with moderate to severe atopic dermatitis	Reduced disease severity, improved quality of life	[155]
18.	Pomegranate juice	Anthocyanins/flavonoids	Punicalagin, anthocyanins	Randomised, double blind, placebo-controlled trial	Overweight/obese individuals	Modulated gut microbiota, increased beneficial metabolites	[156]
19.	Citrus fruits	Flavanones	Hesperidin, naringenin	Randomised controlled trial	Prediabetic patients on metformin	Improved glycaemic control and metabolic parameters	[157]

## 7. Future Perspectives

Despite growing evidence identifying dietary flavonoids as microbiome-responsive modulators of gut–liver axis and hepatic redox signalling, significant translational challenges remain. Profound inter-individual variability in gut microbiome composition generates heterogeneous flavonoid metabolism and inconsistent hepatic outcomes, complicating dose standardisation and limiting efficacy prediction. Concurrently, the lack of harmonised multi-omics infrastructures, including differences in sampling, data integration, analytical platforms, and interpretation, restricts cross-study comparability and system-level mechanistic resolution. These scientific limitations arise from regulatory frameworks for functional foods. They have not evolved to accommodate microbiome-dependent bioactivity claims. Looking ahead, artificial intelligence-assisted integrations of multi-omics datasets offer a transformative opportunity to decode complex flavonoid microbiome–host interactions and advance precision nutrition approaches. The development of flavonoid-derived postbiotics through controlled microbial biotransformation represents a promising avenue for achieving consistent biological effects independent of variations in the microbiome. Ultimately, embedding these innovations within a One Health-oriented dietary framework that considers environmental sustainability, food systems, and human metabolic health may ultimately advance flavonoid-based interventions as evidence-driven, multi-target redox therapies for chronic liver disease.

Future advancements of flavonoids as clinically relevant therapeutic agents depend on progress in pharmacokinetics and the formulation of technology for liver disease. Enhancing their oral absorption, ensuring efficient targeted delivery to hepatic tissue, and developing a controlled or sustained release system, using nanocarriers, prodrug strategies, and other modern delivery platforms, could help to translate the preclinical results into reliable patient outcomes. The development of a clear mechanistic understanding is a key objective. Different flavonoids influence various molecular and cellular pathways, including metabolic regulation, immune signalling, oxidative stress responses, and hepatic stellate cell activation. Identifying which compounds target which mechanisms will enable their rational alignment with particular liver disease subtypes such as viral hepatitis, metabolic dysfunction-associated steatotic liver disease (MASLD), cholestatic disorders, and fibrosis-dominant conditions. Substantial progress in this field will also require high-quality clinical evidence. It is important to have large-scale, well-designed, multicentre randomised controlled trials employing standardised flavonoid formulations and validated clinical endpoints. This includes liver stiffness assessment by elastography, hepatic fat quantification using MRI-proton density fat fraction and biopsy for histological evaluation. Such studies are essential to determine true therapeutic benefits beyond supportive roles. The comprehensive evaluation of long-term safety is equally important, as many individuals with liver disease are prescribed multiple medications. There should be systematic monitoring of potential herb–drug interactions. The implementation of structured pharmacovigilance systems will be necessary to ensure safe incorporation of flavonoid-based interventions into standard clinical practice.

## 8. Conclusions

Flavonoids are emerging as promising naturally derived hepatoprotective agents that safeguard the liver by reducing fibrosis, inflammation, oxidative stress, and metabolic dysregulation. Preliminary clinical findings report improvements in MASLD/NAFLD when flavonoids are combined with lifestyle modifications. Despite these findings, clinical translation is limited by challenges such as formulation inconsistencies, poor and variable bioavailability, and a lack of large-scale, rigorously designed clinical trials. Preclinical studies demonstrate that compounds such as quercetin, silymarin, naringenin, and catechins significantly reduce indicators of hepatic injury. Progress in developing targeted and sustained drug delivery systems, along with a clear understanding of dose–response relationships and mechanism-specific effects, will be crucial to overcoming these limitations. Addressing these gaps may help to translate preclinical benefits into consistent clinical outcomes. With such advancements, flavonoids have the potential to become valuable adjunct therapies for the management of diverse liver disorders. Dietary flavonoids enhance liver health by interacting with gut microbes, rather than direct absorption by the body. Flavonoids convert into bioactive metabolites in the gut microbiome that regulate gut barrier integrity, bile acid homeostasis, and immune signalling, and support normal liver metabolism. Multi-omics evidence indicates that liver diseases arise from complex interactions between the gut microbiome, diet, and environmental factors rather than from hepatic damage alone. However, translation into clinical practice remains constrained by formulation variability, bioavailability limitations, and large-scale clinical trials. Future progress relies on the biomarker-driven therapeutic frameworks and the development of targeted delivery systems, precision nutrition models, and integrative system biology approaches to fully translate flavonoid-mediated redox modulation into effective adjunct treatments for chronic liver disease.

## Data Availability

No new data were created or analysed in this study. Data sharing does not apply to this article.
